# Supplementary material for: Uniquely preserved gut contents illuminate trilobite palaeophysiology
Source: Nature. 2023 Sep 27;622(7983):545–51. doi: 10.1038/s41586-023-06567-7 (PMC10584673; doi:10.1038/s41586-023-06567-7)
Supplement: Supplementary file 1 — Supplementary discussion and references. [file 41586_2023_6567_MOESM1_ESM.pdf]

nature portfolio

https://doi.org/10.1038/s41586-023-06567-7

Supplementary information

Uniquely preserved gut contents illuminate trilobite palaeophysiology

In the format provided by the authors and unedited

Geological settings

The studied fossil was discovered in a siliceous nodule weathered out from the Šárka Formation. This Middle Ordovician unit of early to middle Darriwilian age (latest Arenigian to Oretanian in the regional scale of the European Variscides) is typified by a prevailing uniform sedimentation of grey to dark grey shale. The Šárka Formation is a lithostratigraphic unit in the volcanosedimentary infill of the Prague Basin exposed in central Bohemia (Czech Republic). The basin was an Early Palaeozoic, Ordovician to mid Devonian, depression in the outer shelf of Gondwana in high latitudes of the southern hemisphere. Thus, its early development including the lifetime of the studied fossil was in cool realm conditions.
Levels with siliceous nodules enclosed inside the section are typical for the Šárka Formation. The nodules are very resistant and concentrate in the soil after the denudation of the mother rock. Subsequently, they are periodically reworked by farming and appear on the surface. The nodules have been popular objects of fossil hunters since their discovery in 1855. They have an early diagenetic origin and contain undeformed 3D fossils in contrast with the surrounding shale where the fossils are more or less flattened. On the other hand, typically no shell (with rare exceptions) is present in the nodules. The fossils are preserved as internal and external moulds with free space in between reflecting the dissolved shell.

The fossil associations of the Šárka Formation are among the most diversified and richest in the Bohemian Ordovician. They are composed of more than 150 species[40], about fifty of them are trilobites, and about forty of all species occur frequently. Brachiopods, bivalves, gastropods, ostracods, hyolithids and echinoderms are very abundant; cephalopods are abundant; graptolites and phyllocarids are abundant locally; conulariids, machaeridans, monoplacophorans and rostroconchs are infrequent. The energy sources were rich in the communities of the Prague Basin at that time because of a wide, variable and abundant food supply. It included not only shelly taxa preserved in the fossil associations but also soft-

bodied animals indicated through ichnofossils. Microplankton and algae should be also considered. Therefore, there is also a variety of food strategies among the above-mentioned components of communities such as filter feeders, scavengers or predators (e.g.[41-42]).

The studied fossil, a complete specimen of the trilobite *Bohemolichas incola* (Barrande, 1872)[43], is unique even among the specimens found in the nodules. It was discovered in one of the most famous localities, in the field near the hamlet called Díly, north of Rokycany (1.8 km north-east of the church in Rokycany; 15.5 km east of the Pilsner Urquell brewery in Plzeň, the capital of beer) in the south-western part of the denudation relic of the Prague Basin.

Studied material

Bohemolichas incola (Barrande, 1872) is a trilobite species occurring infrequently in the Šárka Formation. It is usually discovered fragmented, as separate cephala, cranidia, hypostomes, or pygidia. Articulated fragments containing parts of thorax are rare, complete exoskeletons are exceptional. The best specimen, housed in the Museum of Dr. B. Horák in Rokycany under the inventory number 8, is preserved with fine details. The damage of pleura on the posterior left side of the thorax (Fig. 1a) was caused by the opening of the nodule by hammer. The surface of its rachis on the internal mould is peeled off in several sections providing views below the internal surface of the exoskeleton. The specimen was initially inspected with micro-CT, which confirmed the existence of objects situated along the intestine without sufficient details. The subsequent high-resolution scanning at beamline ID 19 of the European Synchrotron Radiation Facility (ESRF) in Grenoble allowed a precise 3D reconstruction of the exoskeleton and associated structures. The specimen represents a young holaspid due to its small size. The dimensions of specimens of *B. incola* in the collections of

the Museum of Dr. B. Horák in Rokycany and the National Museum in Prague show that typical adults of this species are usually twice as large.

Remarks to the digestive tract

The digestive tract can be reconstructed as follows: from the unpreserved mouth and oesophagus supposedly located near the posterior margin of the hypostome it continued to the antero-ventral ventriculus situated dorsally to the anterior lobe of the hypostome. The following antero-dorsal prolongation formed a slightly narrower, curved segment interconnecting the antero-ventral and dorsal ventriculi (i.e. the two-chamber stomach). The latter was a large, sacciform organ positioned below the middle and posterior parts of the glabella representing the anterior beginning of a simple straight remaining portion of the alimentary canal. Similar J-shaped morphology of the digestive tract has been reported in other trilobites[44]. It was comprised mainly of the thick or extensible gut extended along the entire length of the thoracic and pygidial axes.

The fragments inside the digestive tract of *B. incola* are arranged in clusters without any apparent orientation. However, planar fragments near the outer limit of the clusters (originally along the inner surface of the intestine) frequently tend to parallel the outline. Each cluster, i.e. segment of the intestine, is a mixture of fragments different in size, character and origin. The clusters of indigestible remains formed faecal pellets. The deflection of the sixth thoracic segment mirrors a distinct change in the gut content and a noticeable decrease of its width (Figs. 1c and 2a; Extended Data Fig. 1), indicated by a limit between two faecal pellets. It may reflect different food sources and feeding periods but also different feeding intensity. The faecal pellets could have been dispersed immediately after excretion, lay exposed on the sea bottom for some time, or preserved whole if covered with sediment. The presence of large particles, their size range, density, and shape anisotropy evoke a probability of the former

case. Discovery of no corresponding coprolites in any locality of the Šárka Formation can, however, be caused by the absence of *in situ* habitats of the studied species. A strongly prevailing fragmentation and only a few complete specimens found over more than 160 years of intensive collecting of the 'Rokycany balls' indicate an allochthonous origin and are good evidence that *Bohemolichas incola* most probably lived in different habitats rather than in those represented by facies in exposures of the Šárka Formation and the nodule concentrations.

Taphonomical aspects

As the nodule was found loose in the soil, its original orientation in the section is unknown. We can infer the original position from two consecutive taphonomic processes that presumably affected the trilobite carcass. First a compression of the dead organism in a still soft substrate immediately after burial, followed by the decay of tissues in an already forming nodule, i.e. in a solid matter produced by silicification of the muddy substrate at a very shallow depth below the sea bottom. The dislocation of the hypostome and the intestine pressed to the internal surface of the exoskeleton, both shifted in dorsal direction, indicate that the trilobite was buried with its ventral side up (i.e. in a concave position). Both deformations are logically explained to be caused by gravitational collapse under pressure of the soft sediment rather than the pressure of compaction or the influence of decay gases in an opposite (convex) orientation of the exoskeleton. The presumed position is supported by the orientation of the ichnofossils reaching the carcass from the surface rather than from the deeper bottom sediment. The deformation, which postero-dorso-sinistrally shifted the hypostome, slightly compressed both ventriculi and their content, lead also to a prominent sinistral deflection of the anterior interconnection of the ventral and dorsal ventriculi. Based on the above deformations the force acted from the antero-ventro-dextral direction, apparently affecting the

whole body due to the selective compression of body sides. It resulted in the dextral off-axis course of the gut; the hindgut suffered the maximum shift while its anal terminal is almost in its original place. Apparently, the right side of the body was also more affected, because it is notably more intensively targeted by ichnofossils than the left side (Fig. 4c).

There is a substantial difference between the preservation in the nodules and the surrounding shales[45]. After the formation of the nodule, there were no further deformations or collapses of cavities left after the decayed tissues. Therefore, the cavities are preserved and filled by the "crumble", i.e. fossilized products of bacterial activity on the decayed tissues. The tissue decay was happening in cavities entombed in solid matter, which is why their shape did not change further and reflects the original shape of the cavities at the time when the nodule was formed. However, it does not necessarily reflect the morphology of the decayed organs. This process apparently happened very early after the specimen was buried, which is also supported by the behaviour of the scavengers (see chapter Post-mortem events and below).

Narrow and simple burrows are associated with the trilobite exoskeleton. Most of them are simple vertical tunnels penetrating perpendicularly to the body plane toward the ventral side of the trilobite carcass. They are randomly distributed in space reaching the pleura of the thorax where they terminate; several burrows point to the right side of the cephalon and left side of the pygidium. They mostly occur lateralward, including often targeted pleural spines; their concentration notably decreases toward the axis. The number of burrows targeting pleural spines also indicates the decaying tissue in the duplicature of the trilobite exoskeleton (discussed by Kraft and colleagues[12]). Besides the linear to simply bent tunnels there is a prominent cluster of densely arranged burrows in the anterior part of the glabella. The dense burrows in this cluster are concentrated in two lateral subclusters interconnected by at least five transverse, almost straight tunnels.

Compared with the topology of organs in other arthropods there are two candidates for the paired organ composed of two lobes in the anterior most part of the cephalon where the successful individual found a soft undecayed nutrient target. If we consider the brain, the burrow sub-clusters would be oversized. Also, the position of the brain lobes near the base of the antennae but in front of the supposed position of the optic lobes, could be explained only by a strange topology modification of the protocerebrum and the deutocerebrum. Therefore, glands (secreting hormones rather than enzymes) are more probable organs to be targeted by scavengers in the anterior of the trilobite glabella. The position is comparable with the antennal (green) glands of decapod malacostracans. An analogous organ is highly probable in lichid trilobites. It also represents a nutrient target that would not have to decay in order to be fed upon by a weak mouth apparatus (as opposed to, for example, tough muscle tissue, tendons or epidermis).

Value of the exceptional single specimen

Our knowledge about trophic strategies in a number of fossil taxa is quite good based on their functional morphology. Especially in taxa with modern relatives, which enable actualistic comparisons and whose life strategies can be inferred with high probability. Direct evidence, such as exceptional discoveries of food remains preserved inside the intestine, is rare. Such fossils testify not only the trophic strategy but also the food composition. Coprolites or injuries can have the same value but their producer is often dubious.

The digestive tract of trilobites was studied directly in several species[4]. It is usually preserved as low to full relief casts due to the early mineralization (see overview by Fatka and colleagues[46]) or is considered as evidence of a detritus-feeding habit[47]. The precise composition of the trilobite diet has only been estimated without direct evidence.

Among all feeding habits, the one with the highest potential to be identified is unfortunately quite uncommon – a non-selective feeder related to durophagy. Shell fragments present in the intestine in the moment when the animal was buried have a potential to survive all taphonomical and fossilization processes. This feeding habit is known from the fossil record in various groups including the Early Palaeozoic arthropods[17]. It has never been documented in trilobites. As this group did not possess mineralised oral elements for crushing shells, it was not considered to represent a typical durophagous feeder. This discovery means that at least some trilobites, along with related arthropods[17], had a similar feeding strategy in the Early Palaeozoic ecosystems as durophagous decapod crustaceans have in modern ecosystems. Even though lacking the strongly calcified appendages, which enabled this malacostracan clade to spread the feeding strategy widely since the Upper Cretaceous[48], trilobites represent a functional analogy occurring some 250 million years earlier.

The studied specimen captures two interconnected but discrete moments frozen in one nodule. One points to the complex digestive physiology related to a specific overfeeding activity in the life cycle of the trilobite. The other documents the post mortem processes reflected through a snapshot of constrained targeting of scavengers feeding on its corpse.

References

40. Havlíček, V. & Vaněk, J. The biostratigraphy of the Ordovician of Bohemia. *Sborník geologických věd, Paleontologie* **8**, 7–69 (1966).
41. Budil, P., Kraft, P., Kraft, J. & Fatka, O. Faunal associations of the Šárka Formation (Middle Ordovician, Darriwilian, Prague Basin, Czech Republic). *Acta Palaeontol. Sinica* **46**, 64–70 (2007).

42. Polechová, M. Bivalves from the Middle Ordovician Šárka Formation (Prague Basin, Czech Republic). *Bull. Geosci.* **88**, 427–461 (2013).
43. Barrande, J. *Systême silurien du centre de la Bohême. 1ère Partie: Recherches Paléontologiques. Supplément au Vol. I. Trilobites, Crustacés divers et Poissons* (Privately published, Prague and Paris, 1872).
44. Lerosey-Aubril, R. et al. Controls on gut phosphatisation: the trilobites from the Weeks Formation Lagerstätte (Cambrian; Utah). *PLoS ONE* **7**, e32934 (2012).
45. Kraft, P. & Bruthansová, J. Preservation of fossils in the Šárka Formation (Darriwilian, Czech Republic). *Est. J. Earth Sci.* **72**, 136 (2023).
46. Fatka, O., Lerosey-Aubril, R., Budil, P. & Rak, Š. Fossilised guts in trilobites from the Upper Ordovician Letná Formation (Prague Basin, Czech Republic). *Bull. Geosci.* **88**, 95–104 (2013).
47. Zhu, X., Lerosey-Aubril, R. & Esteve, J. Gut content fossilization and evidence for detritus feeding habits in an enrolled trilobite from the Cambrian of China. *Lethaia* **47**, 66–76 (2014).
48. Schweitzer, C. E. & Feldmann, R. M. The Decapoda (Crustacea) as predators on Mollusca through geologic time. *Palaios* **25**, 167–182 (2010).
